# Cortical volume alteration in the superior parietal region mediates the relationship between childhood abuse and PTSD avoidance symptoms: A complementary multimodal neuroimaging study

**DOI:** 10.1016/j.ynstr.2023.100586

**Published:** 2023-11-07

**Authors:** Richard Okyere Nkrumah, Claudius von Schröder, Traute Demirakca, Christian Schmahl, Gabriele Ende

**Affiliations:** a Department of Neuroimaging, Central Institute of Mental Health, Medical Faculty Mannheim, Heidelberg University, 68159, Mannheim, Germany; b Department of Psychosomatic Medicine & Psychotherapy, Central Institute of Mental Health, Medical Faculty Mannheim, Heidelberg University, 68159, Mannheim, Germany

**Keywords:** Adverse childhood experiences, Child abuse, Post-traumatic stress symptoms, Cortical morphology, White-matter tractography

## Abstract

**Background:**

Adverse childhood experiences (ACE), which can be separated into abuse and neglect, contribute to the development of post-traumatic stress symptoms (PTSS). However, which brain structures are mainly affected by ACE as well as the mediating role these brain structures play in ACE and PTSS relationship are still being investigated. The current study tested the effect of ACE on brain structure and investigated the latter's mediating role in ACE-PTSS relationship.

**Methods:**

A total of 78 adults with self-reported ACE were included in this study. Participants completed the childhood trauma questionnaire (CTQ) and a Posttraumatic Stress Disorder Checklist for DSM-5 (PCL-5) to ascertain ACE history and PTSS, respectively. T1w images and diffusion MRI scans were then acquired to assess cortical morphometry and white matter (WM) integrity in fibre tracts connecting key areas where ACE-related cortical volume alterations were observed.

**Results:**

The combined effect of ACE was negatively associated with total grey matter volume and local cortical area in the right superior parietal region (rSP). Childhood abuse was negatively related to right superior parietal volume after controlling for neglect and overall psychological burden. The right superior parietal volume significantly mediated the relationship between childhood abuse and avoidance-related PTSS. Post-hoc analyses showed that the indirect relation was subsequently moderated by dissociative symptoms. Lastly, a complementary examination of the WM tracts connected to abuse-associated cortical GM regions shows that abuse was negatively related to the normalised fibre density of WM tracts connected to the right superior parietal region.

**Conclusion:**

We provide multimodal structural evidence that ACE in the first years of life is related to alterations in the right superior brain region, which plays a crucial role in spatial processing and attentional functioning. Additionally, we highlight that the cortical volume alteration in this region may play a role in explaining the relationship between childhood abuse and avoidance symptoms.

## 1 Introduction

Adverse childhood experiences (ACE) are associated with higher rates of psychiatric disorders later in life (Hailes et al., 2019), and include sexual, physical or emotional abuse and/or neglect experiences. Recent conceptualizations of ACE comprise two dimensional subtypes(DS): abuse and neglect (McLaughlin et al., 2019; Sheridan and McLaughlin, 2014). Abuse involves the presence of an unexpected experience that poses a significant threat of harm to the child, such as sexual, physical, or emotional harm. Neglect, which includes physical and emotional deprivation during childhood, is characterised by a lack of expected environmental inputs, specifically a lack of expected cognitive and social inputs. The frequency and consequences of abuse and neglect were investigated in a 2531-person German sample. Almost half of the sample reported at least one form of abuse and/or neglect and were prone to psychosocial problems involving life satisfaction, psychopathology, and interpersonal aggression (Witt et al., 2019). Consequences of ACE include major depressive disorder, post-traumatic stress disorder (PTSD), borderline personality disorder, attention deficit hyperactivity disorder (ADHD), bipolar disorder and elevated symptom levels of depression, anxiety and dissociative symptoms (Herzog and Schmahl, 2018; Seitz et al., 2022). The general consensus is that childhood abuse and neglect can result in severe developmental problems that are interpersonal, enduring, co-occurring, and linked to high rates of PTSD symptoms (De Bellis and Zisk, 2014).

Evidence shows that ACE influences neural development, leading to changes in brain structure and consequently its function. Several neuroimaging studies on the effects of ACE show that the orbitofrontal cortex (OFC), amygdala, hippocampus and thalamic regions, which are part of the limbic system and play a role in survival behaviour such as feeding and reproduction, and emotional responses, as well as parietal regions including the superior parietal lobe (SPL), are altered in individuals with ACE (McQuaid et al., 2019; Pollok et al., 2022). The SPL forms part of the frontal parietal network (FPN) and also receives input from the thalamus through the medial route of the dorsal visual stream. Therefore, the corollary that both limbic and SPL regions are affected by ACE provides useful information that could be further investigated in future research (Gamberini et al., 2021; McLaughlin et al., 2019). Recent meta-analyses also found ACE to affect cortical thinning in the right medial cingulate cortex and GM volume reduction in the left supplementary motor area (Yang et al., 2023). Following the consistent account of the combined effect of ACE on brain structure, the DS of childhood adversity—abuse and neglect—appear to affect brain structure differently. There is strong evidence that abuse alters the structure of regions that underlie attentional functioning, emotional memories, and inhibitory control, including the hippocampus and regions in the anterior part of the FPN such as the dorsolateral prefrontal cortex(Hart and Rubia, 2012). Neglect, on the other hand, has been shown to alter parts of the orbitofrontal, superior temporal and rostalmiddle frontal gyri, which are involved in the anticipation and receiving of rewards, as well as regions in the posterior part of the FPN such as the SPL (Lim et al., 2014; Mackes et al., 2020). For anatomical and functional details of FPN, please see (Budisavljevic et al., 2017; Marek and Dosenbach, 2018; Parlatini et al., 2017; Thomas Yeo et al., 2011). Some alterations in the amygdala and hippocampus, for example, have been associated with both abuse and neglect. There are, however, some limitations as to how ACE and its subtypes have previously been investigated. Importantly, comorbid mental disorders have not been adequately controlled, which makes it difficult to disentangle which of the effects are due to abuse and/or neglect, or the associated mental conditions, or a combination or interaction of all.

PTSD is a mental health condition that can develop after a person experiences a traumatic event (or a sequence of reoccurring events) such as ACE. Post-traumatic stress symptoms (PTSS) include persistent re-experiencing of the trauma, avoidance of trauma-related circumstances, hyperarousal, and negative alterations in mood and cognition lasting more than a month after experiencing a traumatic event that threatens one's life or bodily integrity. The persistence of PTSS following ACE and its effects on the brain have been documented elsewhere (Siehl et al., 2022; Wang et al., 2021; Xie et al., 2022). For example, cortical alterations in the SPL have previously been negatively associated with PTSS and childhood neglect (Edmiston, 2011; McLaughlin et al., 2014, 2019; Tan et al., 2013). Additionally, correlations between subcortical brain volumes such as the hippocampus and thalamus with ACE and PTSS have previously been reported (Xie et al., 2018, 2022). The findings suggest ACE is negatively associated with thalamic volume post-trauma, which, in turn, is inversely associated with PTSS. Despite this insightful evidence, no study has tested the effects of ACE on cortical morphology while exploring their indirect effect on PTSS, notwithstanding recent ACE-thalamic-PTSS findings and the effect of both ACE and PTSS on some cortical regions such as the SPL.

Extant literature supports the relationship between ACE and white matter alterations measured by diffusion MRI. Over the years, voxel-averaged diffusion quantitative measures like fractional anisotropy (FA), mean diffusivity (MD) and axial diffusivity (AD) have been related to ACE using Tract-Based Spatial Statistics (TBSS) (Lim et al., 2019). Despite these findings, quantitative measures based on averaging voxels are not fibre-specific and may have limited interpretability because most WM voxels contain contributions from multiple fibre populations (commonly referred to as crossing fibres) (Raffelt et al., 2017). Recent advanced 3D DTI fibre tractography provides fibre measures that can be used as the basis for quantitatively assessing the microstructure of specific white matter tracts in mental health studies. For example, the number of fibres indicates the total number of axons in the specific white matter region, while fibre density provides more precise information on the microstructural integrity of a WM tract. These measures are probably more sensitive to certain pathologies, are more directly interpretable, and provide a basis for investigating macroscopic intra-axonal WM volume of biological significance (Riffert et al., 2014). Since certain GM regions are also altered by ACE, it is crucial to consider the structure of the WM regions connected to disease-associated cortical GM regions in order to understand the structural brain alterations associated with mental disorders. This is what we term here "complementary multimodal neuroimaging” i.e., where one neuroimaging modality complements the other, thereby allowing us to shed more light on a wide range of structural brain alterations related to a mental trait.

The scope of the current work was to investigate the effect of ACE on grey matter and adjoining white matter connections. We used a comprehensive approach to first examine the relationship between ACE and total grey matter volume (TGV). Then we tested whether any changes persisted after covarying for potential confounders such as sex, age, estimated Total Intracranial Volume (eTIV), and overall psychological burden. The links between ACE and localised alterations in cortical volume, surface area, and thickness were then explored. We hypothesised that ACE would be negatively related to TGV and that local cortical alterations in several limbic and FPN regions, as mentioned above, would show a negative relationship with ACE after controlling for overall psychological burden. We also aimed to identify brain morphometry associated with abuse when controlling for neglect (and vice versa) and overall psychological burden. Based on previous literature (Morey et al., 2016), we hypothesised that abuse would be negatively associated with cortical alterations in the FPN, including the SPL. Similarly, we hypothesised that neglect would be negatively associated with alterations in the superior temporal and rostral middle frontal gyri. In addition, we investigated the mediating role of ACE-related cortical volume alterations in the relationship between ACEs and PTSS. More specifically, given that previous literature supports the mediating role of subcortical regions such as the thalamus volume in the ACE-PTSD relationship, we sought to confirm if ACE-related cortical volume alterations in our sample mediate the relationship between ACE and 10.13039/501100004232PTSS. We hypothesised that ACE-related cortical volume alterations would be an important aspect of any explanation of how ACE lead to adult PTSS. Lastly, an exploratory complementary analysis using the number of fibres, normalised fibre density, average fibre length, and mean FA of WM tracts connected to local cortical ACE-related volumetric alterations would help shed more light on the diverse structural brain alterations related to ACE.

## 2 Methods

### 2.1 Participants

Eighty participants with self-reported ACE and living in Germany were recruited for the current study. Inclusion criteria for the study were any type of abuse (physical, emotional, and sexual) and/or neglect (emotional and physical) experienced before the age of eighteen. Exclusion criteria included any kind of metal implant, pregnancy, traumatic brain injury, claustrophobia, psychosis, or any form of neuropsychological disorder. Two female participants were excluded at the analysis stage, one due to abnormal brain structure and the other due to an acquisition error in diffusion MRI data, leaving a total of N = 78. A summary of the demographics and psychological measures at the time of assessment is shown in Table 1. The study was approved by the Ethics board of the Medical Faculty Mannheim at Heidelberg University, Germany, and was conducted in accordance with the Helsinki Declaration at the Central Institute of Mental Health in Mannheim. All participants gave written informed consent.

**Table 1:** Descriptive statistics for demographics and psychopathology variables.

Variable	Mean (SD)	Range
Age	31.628 (10.790)	18–59
Sex (female)	65 (83%)	
ACE (CTQ total)	62.538 (19.944)	32.00–117.00
Abuse (CTQ abuse)	26.628 (8.590)	10.00–46.00
Neglect (CTQ neglect)	35.910 (13.167)	17.00–71.00
Psychological burden (BSI total)	0.913 (0.617)	0.06–2.55
Dissociation symptoms (German version of the Dissociative Experience Scale (FDS))	14.158 (12.338)	0.23–55.91
PCL-5	28.090 (17.390)	0.00–69.00
PTSS (PCL- sub scales)		
• Reexperiencing	6.256 (4.453)	0.00–19.00
• Avoidance	3.731 (2.597)	0.00–8.00
• Negative alterations in cognition and mood	10.77 (7.058)	0.00–28.00
• Hyper arousal	8.026 (5.871)	0.00–21.00

Note: N = 78; CTQ total = total score of Childhood Trauma Questionnaire; CTQ abuse = sum score of all abuse subtypes of CTQ; CTQ neglect = sum score of all neglect subtypes of CTQ; BSI total = Global Severity Index of Brief Symptom Inventory (BSI); PCL-5 = Posttraumatic Stress Disorder Checklist for DSM-5.

### 2.2 Procedure

See supplementary information on method for details on the study procedure.

### 2.3 Measures

ACE severity was quantified using the sum of individual sub-types of ACE from the Childhood Trauma Questionnaire (CTQ). A detailed report on the CTQ has been reported in prior literature (Thombs et al., 2007). The CTQ consists of five questions for each type of exposure, and each question prompts participants to rate a particular event on a scale ranging from "Never True" to "Very Often True". Here, we calculated the abuse severity score as the sum of all abuse subtypes of the CTQ (i.e., sexual, physical, and emotional abuse), the neglect severity score consisted of the sum of all neglect subtypes of the CTQ (i.e., emotional & physical neglect) and the combined ACE (CTQ total) was calculated as the sum of abuse and neglect scores.

Overall psychological burden was accessed using the self-report Brief Symptom Inventory (BSI) to identify relevant psychosocial symptoms in our sample. The BSI includes 53 items that cover nine symptom dimensions: depression, anxiety, phobic anxiety, somatization, paranoid ideation, interpersonal sensitivity, obsession-compulsion, psychoticism, and hostility. Items are scored on a 5-point Likert scale ranging from 0 (not at all) to 4 (extremely). The Global Severity Index was calculated by adding the sums of the nine symptom dimensions plus the four additional items that were not included in any of the dimensional scores and dividing by the total number of items to which the individual responded, this score was used to assess current or past symptomatology (BSI total).

The PTSD symptom severity was assessed using the Posttraumatic Stress Disorder Checklist for DSM-5 (PCL-5), which is a self-report measure that corresponds to each of the 20 core DSM-5 PTSD symptoms and asks respondents to rate how much each symptom has bothered them in the past month, scoring responses on a Likert scale ranging from 0 (not at all) to 4 (extremely) (Blevins et al., 2015). Symptoms are classified into four domains in accordance with the DSM-5 criteria for PTSD: re-experiencing, avoidance, negative changes in cognition and mood, and hyperarousal, with total PTSS severity score ranging from 0 to 80 indicating more severe symptoms. The PCL-5 is regarded as the "benchmark" self-report measure of PTSD symptom severity, with strong test-retest reliability (r = 0.84) as well as convergent and discriminant validity (Bovin et al., 2016; Harper et al., 2022; Keane et al., 2014).

The German version of the Dissociative Experience Scale (FDS) was used to assess dissociation symptoms in our study (Spitzer et al., 1998). The FDS is a 44-item self-administered questionnaire which measures the frequency of dissociation symptoms such as absorption, amnesia, and identity disturbances. Items are scored on a scale from 0 (never) to 100 (always). In the FDS, the mean of 44-items is calculated and used as overall dissociative symptoms, and this has been shown to have good reliability and validity based on the DSM definition of dissociation (Spitzer et al., 1998).

### 2.4 Data acquisition

Both T1-weighted (T1w) and diffusion images were acquired using a Siemens Prisma-fit Scanner (Siemens Medical Solutions, Erlangen, Germany) with a 64-channel head coil. A 3-D magnetisation-prepared rapid-acquisition gradient echo (MPRAGE; T1-weighted contrast, Echo Time (TE): 2.01 ms; Repetition Time (TR): 2000 ms; Inversion time (TI): 900ms; FA = 9°; FOV: 256 × 256 mm; number of slices 192, voxel size 1 × 1 × 1 mm³) and a double spin-echo echo-planar imaging (EPI) sequence (82 vol, 3 at b = 0 and 79 at b = 1000 s/mm^2^, TR = 8400 ms, TE = 86 ms, matrix = 128 × 128; number of slices 64, and voxel size = 2 × 2 × 2 mm^3^) scans were acquired for each participant.

### 2.5 Data processing

Preprocessing for both T1w and diffusion images was performed using Connectome Mapper 3 (CMP; an open-source Phython3 neuroimaging processing pipeline software developed by the Connectomics Lab, University Hospital of Lausanne (CHUV)). CMP uses a combination of well-known neuroimaging software packages to implement full anatomical and diffusion processing pipelines from raw images (Tourbier et al., 2022). All images were controlled for quality (see supplementary method for details). The preprocessing steps that were used in this study can be seen below.

T1-weighted images were preprocessed, parcellated, and segmented into cortical thickness, surface area, and volume using the FreeSurfer version 6.0.1 recon-all program. An in-depth explanation of the steps used by FreeSurfer's recon-all has previously been described elsewhere (Dale et al., 1999; Fischl et al., 2004). In brief, the white matter and pial surfaces were identified after motion correction, non-uniform intensity normalization and normalization, by creating a mesh around the white matter and pial voxels. Surface-based maps of each individual scan were created using spatial intensity gradients across tissue classes (Desikan et al., 2006). Cortical thickness, surface area, and volume maps were extracted and smoothed with a 10-mm kernel at full width at half maximum (FWHM). FreeSurfer morphometric procedures have been demonstrated to show good test-retest reliability across scanner manufacturers and across field strengths (Reuter et al., 2012). Visual inspection was done to inspect the anatomical accuracy of FreeSurfer's automated parcellations and segmentations.

Denoising and subsequent correction for bias field, eddy currents, and motion correction were performed on all diffusion data using state-of-the-art methods implemented in the MRtrix3 toolbox (J. D. Tournier et al., 2019). Anatomy-constrained probabilistic tractography was performed using the five-tissue-type (5 TT) segmented T1w image and a second-order integration over fibre orientation distributions algorithm on the preprocessed diffusion image to produce an initial tractogram with 10 million streamlines (J.-D. Tournier &, F. Calamante, 2010). The tractogram was filtered using SIFT2 approach: an approach to improve the quantitative nature of whole-brain streamlines reconstructions (Smith et al., 2015). Diffusion measures (i.e., number of fibres, average fibre length, normalised fibre density, and mean FA) touching/emerging from the segmented regions were then extracted for subsequent statistical analyses.

### 2.6 Statistical analysis

#### 2.6.1 ACE relation to total GM volume

To test the first hypothesis, we extracted the total GM volume from the output of recon-all processing to do a preliminary comprehensive regression analysis and to first examine whether ACE (i.e., CTQ total) is associated to total GM volume as hypothesised. We also tested whether the relation persisted after covarying for potential confounders such as age, sex, estimated Total Intracranial Volume (eTIV), and overall psychological burden (i.e., BSI).

#### 2.6.2 Regional cortical alterations following ACE

Whole-brain surface-based analyses were performed using FreeSurfer's glmfit. The general linear model was used to locate all regional cortical alterations in thickness, surface area, and volume that were related to CTQ total for the first hypothesis. This resulted in three models, one for each cortical measure. For the second hypothesis, the effect of subtypes of ACE (i.e., abuse and neglect), regional cortical alterations in relation to ACE subtypes was investigated. First, a simple linear regression was used with either abuse or neglect as variables of interest and age, sex BSI total and neglect or abuse as control variables (6 models in total; 2 variables of interest x 3 cortical measures). Then, a *t*-test was used to investigate the differences between abuse and neglect in the direction of abuse > neglect, because CTQ total has higher correlation to abuse (r_partial_ = 0.929, p < 0.001), compared to neglect (r_partial_ = 0.844, p < 0.001), and controlling for age, sex, and BSI total (one model). All cortical volume analyses were controlled for eTIV, and all results presented here were corrected for multiple comparisons using Monte Carlo simulation with vertexwise threshold P < 0.005 and clusterwise threshold P < 0.05 and in both brain hemispheres. Significant clusters were labelled using Desikan-Killiany atlas.

#### 2.6.3 Mediating role of ACE-related cortical volume alteration in ACE– PTSS relationship

To tackle our third hypothesis, values of significant ACE-related clusters identified in cortical analyses as sensitive to ACE and its sub types were extracted to find out if the significant effects mediate the relationship between ACE and PTSS. The average cortical volume per vertex of each cluster for every participant was multiplied by the number of vertices in the respective clusters to get the total volume per cluster (TVC) for all subjects. This was then used as mediators in the relationship between ACE and PTSS. The bias-corrected CIs and SEs for the mediation effect are reported here using 5000 bootstraps. All mediation analyses were performed using JASP (JASP Team, 2023). As all mediation models were just identified, no model fit indices were computed as previously reported here (Mackes et al., 2020). Lastly, since PCL-5 does not link PTSS to a specific type of trauma, our aim here is to examine the association between ACE and PTSS regardless of whether the cause of the PTSS is due to ACE alone or also due to additional trauma events.

#### 2.6.4 Diffusion measures in WM complements local cortical ACE-related GM volume alterations

To complement the local cortical ACE-related volumetric alterations in GM regions identified in the previous analyses, diffusion measures (i.e., number of fibres, average fibre length, normalised fibre density, and mean FA) in WM pathways that connect to ACE-related GM volume regions were extracted to verify their relation to ACE. Hence, we could further explore the local WM integrity connected to GM regions relation to ACE using regression models. We extracted the diffusion measures touching/emerging from the segmented GM regions in Desikan-Killiany atlas space and correlated diffusion WM measures with TVC from the abuse/neglect subtype analyses. Each model was corrected for multiple comparisons using the false discovery rate q = 0.05. The effect sizes were bootstrapped using 5000 iterations and bias-corrected CIs and SEs were reported.

## 3 Results

### 3.1 Associations between ACE and total GM volume

We observed a negative association between total CTQ score and total GM volume: β = −768.825, t (76) = -2.515 and p = 0.014. This result remained significant after controlling for sex and BSI total (β = −725.517, t (74) = -2.426 and p = 0.018), suggesting that the effects were not simply a reflection of other psychological disorders or sex. Although previous reviews show that BSI captures some form of psychological distress that commonly occurs in the chronic posttraumatic phase (Auxéméry, 2018; Recklitis et al., 2017), the check for multicollinearity shows that the presence of the BSI total variable does not affect our regression analysis (i.e., the VIF of 1.112; also see Supplementary Table 1). We noticed that including age in the model diminishes the effect, i.e., the relationship between total GM volume and CTQ total becomes statistically non-significant (β = −256.252, t (73) = -0.430 and p = 0.379). Also, CTQ total showed no significant relationship with total GM volume when estimated total intracranial volume (eTIV) was controlled for (Supplementary Table 2). Despite these findings, we did control for age in all cortical analyses and additionally for eTIV in cortical volume analysis based on previous literature (Pollok et al., 2022; Voevodskaya et al., 2014). Lastly, since CTQ total and total GM volume were negatively related, all subsequent cortical regional analyses focused on this negative relationship.

### 3.2 Local alterations in cortical structure following ACE

**Fig. 1:**
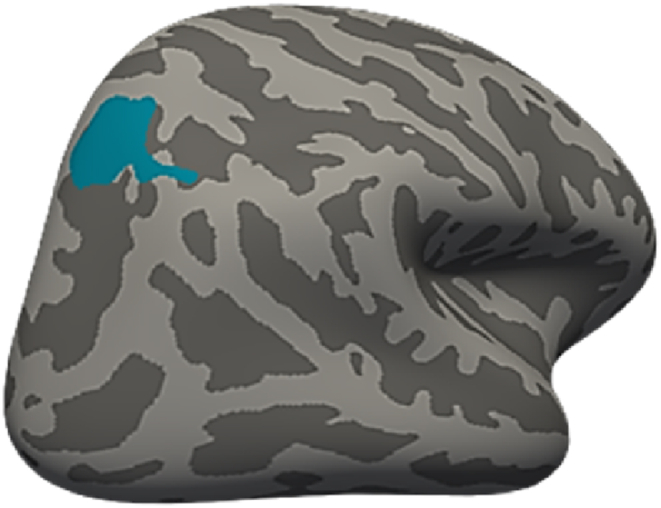
Negative effect of ACE on cortical surface area in right superior parietal region after controlling for overall psychological burden, age, and sex.

Using the whole brain surface-based analysis approach, we identified a cortical area reduction in the right superior parietal area to be related to CTQ total after controlling for overall psychological burden, age, and sex(Fig. 1 and Table 2).

**Table 2:** Cluster showing significant negative relation between CTQ total and cortical surface area.

Cortical Measure	H	Brain region	Size (mm^2^)	MNI coordinate [x y z]	Clusterwise P	Effect size
Area	RH	Superior parietal	694.21	18.9–60.4 54.4	0.0443	−4.0089

Monte Carlo correction for multiple comparisons was applied (clusterwise threshold P < 0.05, vertex-wise threshold P < 0.005). Effect sizes (regression coefficients) were taken from whole brain vertexwise effect size brain maps. H, hemisphere; RH, right hemisphere; LH, left hemisphere.

### 3.3 Effect of abuse and neglect on cortical brain measures

**Fig. 2:**
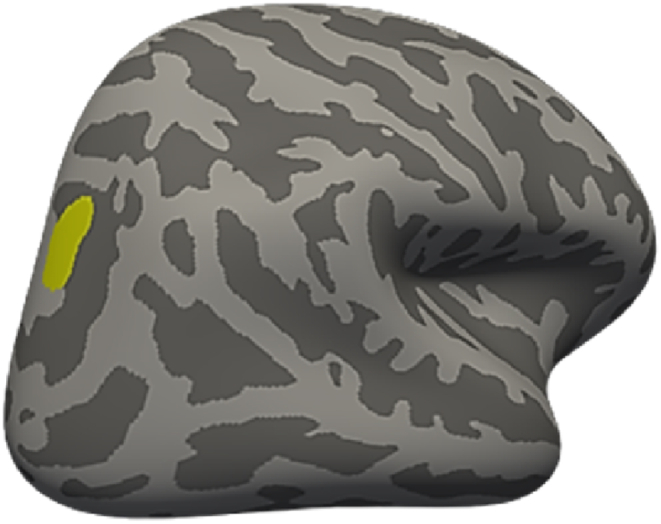
Significant effects of abuse on local cortical volume in the right superior parietal region after controlling for the effects of neglect severity, overall psychological burden, age, eTIV and sex.

We used a simple linear regression with Abuse/Neglect variables of interest and sex, BSI total, age, eTIV and abuse/neglect as control variables. For the differences between abuse and neglect on cortical measures, a *t*-test was used in the direction of Abuse > Neglect, and controlling for overall psychological burden, age, sex and eTIV (for volume). Abuse was significantly negatively related to cortical volume in the right superior parietal region after controlling for neglect, sex, age, eTIV, and overall psychological burden (see Fig. 2 and Table 3 below) No other significant associations were observed between neglect and all cortical measures. Additionally, the *t*-test of abuse > neglect on all cortical measures also showed no significant association.

**Table 3:** Cluster showing a significant negative relation between childhood abuse and cortical volume alteration.

Cortical Measure	H	Brain region	Size (mm^2^)	MNI coordinate [x y z]	Clusterwise P	Effect size
Volume	RH	Superior parietal	369.99	21.6–62.6 37.6	0.0412	−3.5479

Monte Carlo correction for multiple comparisons was applied (clusterwise threshold P < 0.05, vertex-wise threshold P < 0.005). Effect sizes (regression coefficients) were taken from whole brain vertexwise effect size brain maps. H, hemisphere; RH, right hemisphere; LH, left hemisphere.

### 3.4 Does abuse-related cortical volume alteration in the right superior parietal lobe (rSPV) mediate the relation between abuse severity and PTSS severity scores?

**Fig. 3:**
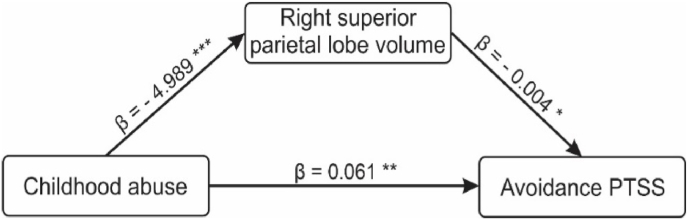
Significant mediation role of abuse-related volume reduction in the right superior parietal lobe in the relationship between the severity of childhood abuse (assessed with the total CTQ abuse severity score) and PTSD avoidance symptoms (Avoidance PTSS; assessed with the PCL avoidance symptomatology). Asterisks indicate the statistical significance of the bootstrapped standardised regression coefficients (***p < 0.001; **p < 0.01; *p < 0.05).

To address this, we used rSPV as a mediator, abuse (assessed with the total CTQ abuse severity score) as a predictor, and all four PTSS severities (assessed with PCL) as outcome variables (giving a total of four mediation models). Each model was deemed significant if the p-value of the total effect was less than the Bonferonni corrected p-value (i.e., p < 0.05/4 = 0.0125). All four models were significant after Bonferroni correction (see supplementary table S4). The direct relationship between childhood abuse and each PTSS dimension was significant (see Table 4). rSPV significantly mediated the relationship between abuse and avoidance PTSS (n = 78, β = 0.021, SE = 0.010, Z = 2.130, 95% CI = [0.004, 0.042], R^2^ = 0.217, p = 0.033). The path plot showing effects is depicted in Fig. 3 below. No other significant rSPV mediation in the other three models was found, even though the total effects of all models were significant (see supplementary tables S3 and S4). Lastly, the path between abuse and rSPV (β = −4.989, p < 0.001), and rSPV and negative changes in cognition and mood PTSS (β = −0.011, p = 0.045) were both significant, but their total indirect effect was insignificant in the abuse-rSPV-PTSS_negative changes in cognition and mood_ model (β = 0.008, p = 0.059) (see supplementary table S3 ).

**Table 4:** Direct relation of abuse and all PTSD symptoms.

							95% Confidence Interval	
			Estimate	Std. Error	z-value	p	Lower	Upper
Abuse	→	INTRU	0.138	0.046	3.000	0.004	0.042	0.224
Abuse	→	AVOID	0.061	0.023	2.636	0.008	0.016	0.109
Abuse	→	COMO	0.236	0.062	3.829	<.001	0.124	0.365
Abuse	→	HYPE	0.198	0.051	3.865	<.001	0.100	0.303

*Note.* INRU = intrusive PTSS, AVOID = avoidance PTSS, COMO = negative changes in cognition and mood PTSS, HYPE = hyperarousal PTSS. Bias-corrected percentile bootstrap confidence intervals. Estimator = Maximum likelihood, Optimization method = NLMINB.

### 3.5 Post-hoc analyses

**Fig. 4:**
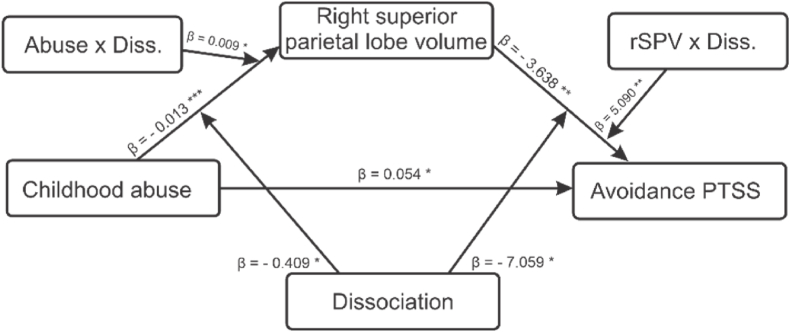
Model diagram showing moderation role of dissociation in the indirect effect of abuse-rSPV- avoidance PTSS relationship. Asterisks indicate statistical significance of the bootstrapped standardized regression coefficients (***p < 0.001; **p < 0.01; *p < 0.05).

**Does the indirect effect in the abuse-rSPV-avoidancePTSS relationship depend on dissociation symptoms?** Several factors, including enhanced memory suppression, developing safety behaviours, and heightened dissociation, contribute to the association between childhood abuse and PTSD symptomatology. Specifically, dissociation is believed to be a coping mechanism for severe trauma experienced during childhood (Kratzer et al., 2018). As post-hoc analyses, we explored whether the significant indirect effect in the abuse -rSPV-avoidance PTSS relationship (mediation analysis in Fig. 3) depends on dissociation in our sample. First, we checked whether dissociation mediates the relationship between the severity of childhood abuse and avoidance PTSS. We found no significant mediation of dissociation in the abuse and avoidance relationship (β = 0.014, SE = 0.008, Z = 1.848, p = 0.065, 95% CI = [0.001 0.030], R^2^ = 0.212) even though the path between abuse and dissociation (β = 0.327, p = 0.003), and the total effect (β = 0.082, SE = 0.020, p < 0.001, 95% CI = [0.082 0.418]) were significant. Then we explored whether dissociation symptoms interact with one or both indirect paths in our main mediation model from Fig. 3. Prior to the analysis and to improve interpretation, we dichotomised the dissociation symptom measure (i.e., FDS score using cut-off 13; Rodewald et al., 2006) in Table 1. The moderated mediation analysis was also performed using lavaan-SEM and is similar to what is implemented in Hayes model 58 (Hayes, 2012) (see Fig. 4 below). Both indirect paths were significant, hence, we subsequently explored the CIs and SEs using bootstrapping (See Table 5).

**Table 5:** Table showing whether dissociative symptoms interact with one or both paths in the mediation model in Fig. 3.

Path	Estimate	Std.Err	95% CI		R^2^	p
			L	H		
SPL volume					0.280	
✓ a1- Abuse	**−0.013**	0.003	−0.020	−0.007		<0.001 *
✓ a2-Dissociation	**−0.409**	0.175	−0.744	−0.065		0.020 *
✓ a3- Abuse * Dissociation	**0.009**	0.004	0.001	0.018		0.036 *
PTSD AVOID					0.346	
✓ b1- rSPV	**−3.638**	1.084	−5.735	−1.401		0.001 *
✓ b2-Dissociation	**−7.059**	2.786	−12.988	−2.050		0.011 *
✓ b3- rSPV * Dissociation	**5.090**	1.737	1.894	8.813		0.003 *

Abuse = childhood abuse, rSPV = right superior lobe volume. Bootstrapping is based on 5000 replicates and the coefficient estimate is based on the percentile of the bootstrap distribution, Std.Err is the standard error, CI is the confidence interval and p is the p-value. Significant paths are highlighted with * in the p-value column.

### 3.6 Diffusion measures in WM tracts touching the right superior parietal lobe complement the abuse-related effects in the brain

Following CTQ abuse-related changes in the right superior parietal volume, the diffusion measures (number of fibres, average fibre length, normalised fibre density and mean FA) within WM tracts touching the right superior parietal region were extracted for all subjects and put into regression models to explore their relationship with abuse severity using Pearson's correlation. The average vertexwise volume in the abuse related rSPL cortical volume alteration was significantly correlated to almost all our diffusion measures (see Table 6).

**Table 6:** Complementary correlation analysis of the diffusion measures in WM tracts touching the right superior parietal lobe with abuse-related cortical volume alterations.

Diffusion Measures	Person's r	95% CI [ Lower Upper ]	p
**Number of fibres**	0.330	0.146 0.499	0.003**
**Average length of fibres**	0.298	0.105 0.481	0.008**
**Normalised fibre density**	−0.241	−0.473 –0.010	0.033*
**Mean FA**	0.266	0.083 0.464	0.019*

Pearson's correlations (r) and CI is the confidence interval based on 5000 replicates and p is the p-value. Since the average vertexwise volume was used (i.e., residuals from cortical analyses) we did not include any control variable at this level. Significant relation was heighted as ***p < 0.001; **p < 0.01; *p < 0.05.

## 4 Discussion

This study provides evidence for the combined ACE severity and abuse subtype effects on brain structure. In a multiple regression analysis, ACE was negatively associated with the total GM volume after controlling for the overall psychological burden and sex. Whole brain analyses showed local cortical area reduction in the right superior parietal region to be associated with ACE. No further significant relationships between the combined ACE severity score and whole brain cortical measures were evident in our sample. As opposed to the cumulative account of childhood adversity, the two dimensional subtypes of adversity (i.e., abuse and neglect) may reflect different underlying dimensions of environmental experience that may have distinct associations with neurodevelopmental processes and also influence emotional, cognitive and neural development (McLaughlin et al., 2019). We found cortical volume alterations in the right superior parietal lobe (rSPL) to be associated with abuse while controlling for neglect, age, sex and eTIV. No further significant relationships were present in the ACE subtype analyses after controlling for overall psychological burden, which is crucial to elucidate the effects of abuse/neglect independently from those associated with mental comorbidities (Pollok et al., 2022). The rSPL forms part of the posterior-FPN and has previously been reported to play a key role in the “top-down” or goal-driven allocation of attention. Cytoarchitectonic research shows that the SPL has a complex, heterogeneous architecture with more than seven sub-regions. The receptor distribution patterns and regional cytoarchitectonic features found three sub-regions in Brodmann (BA) 5 and four in BA 7 (Scheperjans et al., 2008; Scheperjans et al., 2005). Functions of these regions were explored in a resting-state functional MRI study in healthy participants, and the results showed that each of the seven sub-regions was connected to several resting-state networks, with the most consistent connectivity observed with the visual and attention networks (Alahmadi, 2021). Although abnormalities in rSPL has been associated with PTSD, PTSS and maternal stress (McQuaid et al., 2019; Wang et al., 2021), no study that examined the superior parietal cortex structure found childhood trauma-related differences (McLaughlin et al., 2019). Based on these results, it seems likely that our sample gives new insights into the possibility that ACE may, at least in part, be related to cortical alterations in rSPL, whose function is related to visual and attention tasks.

The test of our third hypothesis revealed a significant indirect path in the abuse-rSPL-avoidance PTSS relationship. In our four mediation models and as expected, the direct paths between childhood abuse and all the different PTSS measured by PCL were significantly positive-related. This is an indication that persons with ACE may indeed be more prone to developing PTSS (Kratzer et al., 2018). The only indirect path that remained significant was the abuse-rSPL-avoidance PTSS relationship (see Fig. 3 and also S2). Therefore, the right superior parietal volume significantly mediated the relationship between childhood abuse and avoidance PTSS. Comparing the standardised beta estimates of the indirect path (β_ab_ = 0.021) to the direct path (β_c’_ = 0.061) describes the reduced effect, implying that rSPL volume may explain part of the impact of childhood abuse in producing avoidance PTSS. Since a previous mega-analysis in a large sample found smaller volumes in the rSPL to be related to PTSD, we are adding to this finding that the rSPL may play a role in the development of avoidance symptoms in individuals with a history of severe childhood abuse.

A moderated mediation analysis is used to measure how much a mediated effect changes with different degrees of a moderator. As opposed to a mediation analysis, the evidence for a moderated mediation can be used to support the evidence for a mediation under less stringent confounding condition analyses (Loeys et al., 2016). Our post-hoc analyses gave insights into possible conditional indirect findings in the mediation. Dissociative symptoms, including amnesia, depersonalization, and identity fragmentation, often serve as coping mechanisms for severe trauma experienced during childhood (Brand and Frewen, 2017). Since there is a close relation to attention, the involvement of the rSPL here is of interest. Many authors have emphasised the importance of dissociation in PTSD. Some authors agree that dissociation serves as a dysfunctional coping mechanism that serves to prevent biographical memories from integrating traumatic memories and hence perpetuates avoidance PTSD symptoms (Dalenberg and Carlson, 2012; Kratzer et al., 2018). Starting from the left side of the path plot in Fig. 4, both abuse severity and dissociation were negatively associated to rSPV. Their interaction, however, was positively related to rSPV, which in turn was positively related (i.e., through rSPV and Dissociation interaction; right hand side of Fig. 4) to avoidance PTSS. This is interesting because this relationship could help to explain why persons with both childhood abuse and dissociative symptoms (and high abuse related-rSPL volume alterations) exhibit higher avoidance PTSS as a result of dissociation (Kratzer et al., 2018). Since dissociation can serve as a way to cope with the distressing memories and emotions associated with the childhood abuse, leading to higher levels of avoidance behaviours as a means of managing the traumatic experiences indirectly, our findings support this view via the increase in abuse-related cortical volume in the right superior parietal lobe. This view is additionally supported by closely comparing the beta estimates of the interaction in both indirect paths of the moderated mediation model (i.e., abuse-diss. = 0.009 and rSPV-diss. = 5.090), which show the increase in the conditional effect is mostly explained by alterations in the rSPL in the presence of dissociation.

In our exploratory complementary analysis of WM tracts connected to the GM volume regions, we focused on abuse-related cortical alterations in rSPL volume. We made this choice due to the dimension of the cortical volume measure which makes it biologically comparable to 3D DTI fibre tractography measures. The total volume per cluster from the abuse subtype analyses was correlated to almost all the WM measures (i.e., number of fibres, normalised fibre density, average-fibre length, and mean FA) connecting to abuse-related volume alterations. An increase in abuse-related effect on rSPL volume also increases the number of fibres, average fibre length, and mean FA of the WM tracts connected to rSPL, whereas an increase in abuse-related effect on rSPL volume led to a reduced normalised fibre density of WM tracts connected to rSPL. The former relationship was unexpected because the more the abuse-related effect in rSPL volume increased, we expected all the WM measures to be reduced, to show that childhood abuse to some extent also negatively affects WM tracts connected to rSPL. This might be explained by the fact that these quantitative measures do not account for individual brain size, in contrast to normalised fibre density, which accounts for brain sizes. The normalised fibre density measure is an upgrade of the fibre density measure proposed by Hagmann et al. (2008) to account for individual brain sizes by normalizing the number of fibres connecting two regions by the total number of fibres in the tractogram and additionally, normalizing the surface per volume of the two regions by the total surface per volume of all regions (Daducci et al., 2012; Hagmann et al., 2008; Tourbier et al., 2022). Our findings provide further insight into the structural integrity of the WM tracts connected to the rSPL and affected by childhood abuse. It is noteworthy that not only was the cortical volume negatively associated with abuse, but the abuse-related volume in rSPL was negatively related to the normalised fibre density measure, which accounts for individual brain sizes.

There are some limitations to our study. First, we collected data about ACE using self-reported questionnaires. Thus, there might be a recall bias, as a meta-analysis reported low agreement between prospective and retrospective measures of ACE (Baldwin et al., 2019). However, self-report measures are mostly used in ACE research because they provide a unique window into the subjective experiences of individuals with ACE and allow them to express their feelings, thoughts, and perceptions of the experience. Interestingly, subjective experience of ACE were stronger associated with emotional disorders in adulthood than objective prospective measures (Danese and Widom, 2023), and therefore potentially also to brain alterations. Hence, using self-reported measures in our study is justified as it provides first-hand information about the experience and a contextual understanding of its effects. Second, it is still unclear to what extent pubertal development, malnutrition, prenatal drug exposure, and resilience to co-factors from childhood to adulthood may have influenced our findings since we didn't collect data on this. Hence, not controlling for these factors could be a limitation. Third, despite the positive insights provided by this study's design, mediation and moderated mediation analyses do not infer causality in cross-sectional studies like ours and hence should be cautiously interpreted. Thus, we reiterate that these analyses are exploratory. Despite this limitation, we have tried to ensure the statistical robustness of our findings by implementing bootstrapped confidence intervals as recommended by Edwards and Konold (2020). Fourth, we acknowledge that in a subset of individuals, the PTSS could be due to other traumatic experiences unrelated to ACE such as adult trauma exposure. Because higher ACE was associated with higher PTSS (which takes into account the experience of traumatic events throughout life, i.e., in both childhood and adulthood), our model examines the association between abuse and PTSS regardless of whether the cause of the PTSS is due to ACE alone or also due to additional trauma events. Finally, since the majority of the participants were female (83%), the findings may not generalize well to men. We recommend that future studies should use longitudinal designs to assess changes in adversity over time (i.e., to include adult trauma exposure) and also balance male and female participants in a large sample size to help generalize the results to different samples.

## 5 Conclusion

Our study provides novel perspectives about the association between ACE and brain structure and the mediating role of the right superior parietal volume in the relationship of childhood abuse and PTSD avoidance symptoms. These findings contribute to our understanding of the neural mechanisms underlying the development and maintenance of PTSD symptoms, specifically avoidance symptoms, in individuals with a history of childhood abuse. By examining the role of the superior parietal region, our study provides valuable insights that may inform future research and interventions aimed at treating and preventing PTSD in an ACE population. Furthermore, our findings elucidate the complex interplay between this relationship and dissociative experiences as the later moderated the indirect effect in the abuse-rSPV-avoidance PTSS relationship. These findings underscore the potential long-term impact of childhood trauma on the brain, the role of dissociative symptoms, and the development of avoidance PTSD symptoms. Lastly, the normalised density of the WM tracts connected to the right superior parietal region provides improved information on structural brain alterations in persons with ACE.

## CRediT authorship contribution statement

**Richard Okyere Nkrumah:** Conceptualization, Methodology, Software, Formal analysis, Writing - original draft, Writing - review & editing, Visualization. **Claudius von Schröder:** Methodology, Resources, Writing - review & editing. **Traute Demirakca:** Validation, Writing - review & editing. **Christian Schmahl:** Conceptualization, Writing - review & editing, Supervision. **Gabriele Ende:** Conceptualization, Writing - review & editing, Supervision.

## Funding information

This work was supported by the 10.13039/501100001659Deutsche Forschungsgemeinschaft, Grant Number: GRK2350/1. The funders had no role in study design, data collection and analysis, decision to publish, or preparation of the manuscript.

## Declaration of competing interest

None

## Data Availability

Data will be made available on request.
