# Supplementary material for: Cortical volume alteration in the superior parietal region mediates the relationship between childhood abuse and PTSD avoidance symptoms: A complementary multimodal neuroimaging study
Source: Neurobiol Stress. 2023 Nov 7;28:100586. doi: 10.1016/j.ynstr.2023.100586 (PMC10689271; doi:10.1016/j.ynstr.2023.100586)
Supplement: Multimedia component 1 [file mmc1.docx]

**Supplementary Information for**

Cortical volume alteration in the superior parietal region mediates the relation between the childhood abuse and PTSD avoidance symptoms: a complementary multimodal neuroimaging study.

This PDF file includes:

Supplementary information on the method.

Table S1, S2, S3 and S4

SI References

# **1.0 Method:**

## 1.1 Procedure

## This study is part of a larger, ongoing study that is investigating the effects of ACE on brain structure and function (https://grk2350.de/research-projects/ace-characteristics/). Participants were recruited through distributed flyers, advertisements, and online platforms. It is important to note that psychiatric conditions did not factor into our recruitment criteria. Instead, our study was designed to investigate brain alterations following ACE, not specific to any psychiatric condition. The study protocol consisted of online questionnaires, a diagnostic interview, and MRI scanning sessions. The online questionnaires included the Childhood Trauma Questionnaire (CTQ; Thombs, Lewis, Bernstein, Medrano, & Hatch, 2007), the Brief Symptom Inventory (BSI; Derogatis, 1975), dissociative symptoms (i.e., German version of the Dissociative Experience Scale, (Spitzer et al., 1998)) and the diagnostic session included the Clinical version of the Structured Clinical Interview for DSM-5 (SCID-II; First, Williams, Karg, & Spitzer, 2016). The diagnostic interview sessions were conducted by doctoral students who have received SCID-II training. The diagnoses that were assessed included affective disorders, anxiety disorders, obsessive-compulsive disorder (OCD), post-traumatic stress disorder (PTSD), attention deficit hyperactivity disorder (ADHD), and substance use disorder (SUD). Past and current disorders were diagnosed based on the presence of DSM-5 criteria for each disorder. Psychotic disorders were an exclusion criterion. Table S1 summarises the diagnostics.

| Table S1: Number of participants with SCID diagnostics | | |
| --- | --- | --- |
| Variable | current | past |
| PTSD | 22 | 39 |
| Affective Disorders | 22 | 51 |
| Anxiety and Obsessive Compulsive Disorders | 27 | 36 |
| Somatic symptom and related disorders | 5 | 7 |
| Eating Disorders | 2 | 17 |
| Substance Use Disorders | 3 | 14 |
| ADHD | 2 | 3 |

PTSD = posttraumatic stress disorder; ADHD = attention deficit hyperactivity disorder. Current = current diagnostics based on DSM-5 criteria; past = past diagnostics based on DSM-5 criteria. Participants with missing SCID data = 2.

For the current study, only the lifetime PTSD diagnostics (i.e., current and/or past = 39) were used to correlate with the total PCL score (i.e., PCL-5 from Table 1 in the main text). From the point-biserial correlation, lifetime PTSD and total PCL score were found to be moderately positively correlated, r_pb_(74) =.47, p <.001.

# **2.0 Results:**

2.1 Comprehensive regression analysis between total GM volume and total CTQ score

We examined the potential role of confounders by testing whether they account for the relationship between ACE and GM volume. Statistically taking account of these confounding factors is especially important in order to control for their potential effect in our sample. These variables include age, sex, eTIV, and BSI total, which is the Global Severity Index of the Brief Symptom Inventory (BSI).

| Table S2. Comprehensive regression analysis between total GM volume and total CTQ score. | | | |
| --- | --- | --- | --- |
| Models  (1) (2) (3) | | | |
|  | total GM volume (mm^3^) | total GM volume (mm^3^) | total GM volume (mm^3^) |
| Age | - | - | -1990.694***  (276.577) |
| Sex | - | 59407.766 ***  (15,829.877) | 7988.567^ns^  (8829.914) |
| eTIV | - | - | 0.325***  (0.024) |
| Psychological burden (BSI total) | - | 6058.081 ^ns^  (10113.341) | 1071.723 ^ns^  (4934.821) |
| ACE (CTQ total) | -768.825*  (305.702) | -725.517 *  (299.019) | 57.771^ns^  (158.689) |
| Intercept | 720,615.568  (20054.910) | 702,473.549  (19537) | 249662.170  (38758.965) |
| R^2^  RMSE  p-value | 0.077  55318  0.014 | 0.227  49618.814  < 0.001 | 0.822  24145.284  < 0.001 |
| Collinearity Statistics  (Tolerance / VIF) |  | BSI total = 0.899 / 1.112 | Age = 0.859 / 1.164  BSI total = 0.881 / 1.135  eTIV = 0.949 / 1.054 |

Note: N= 78; CTQ total = total score of Childhood Trauma Questionnaire; eTIV = estimated Total Intracranial Volume; BSI total = overall Psychological burden; RMSE= Root Mean Square Error; VIF= Variance inflation factor. The values in the column of each model represent the unstandardized beta coefficients with their standard error in brackets. Asterisks indicate the statistical significance of the bootstrapped unstandardized regression coefficients (***p < .001; **p < .01; *p < .05; ns=not significant).

2.2 Supplementary results for the mediation models

Supplementary results for the mediating role of abuse-related cortical volume alteration in the abuse-PTSS relationship. Abuse-related cortical volume alteration in the right superior parietal lobe (rSPV) significantly mediated the relationship between abuse and avoidance PTSS. Tables S3 and S4 show the indirect and direct paths of all four mediation models respectively.

| Table S3. Indirect relation in abuse, rSPV and all PTSD symptoms | | | | | | | | | | | | | | | | | | | |
| --- | --- | --- | --- | --- | --- | --- | --- | --- | --- | --- | --- | --- | --- | --- | --- | --- | --- | --- | --- |
|  | | | | | | | | | | | | | | | | **95% Confidence Interval** | | | |
|  |  | |  |  | |  | **Estimate** | | **Std. Error** | | **z-value** | | **p** | | **Lower** | | | **Upper** | |
| Abuse | → |  | rSPV | → |  | AVOID | 0.008 |  | 0.004 |  | 2.291 |  | 0.022 |  | 0.0010 | |  | 0.0150 |  |
| Abuse | → |  | rSPV | → |  | INTRU | 0.006 |  | 0.004 |  | 1.357 |  | 0.175 |  | -0.0030 | |  | 0.0140 |  |
| Abuse | → |  | rSPV | → |  | COMO | 0.008 |  | 0.004 |  | 1.892 |  | 0.059 |  | -0.0003 | |  | 0.0160 |  |
| Abuse | → |  | rSPV | → |  | HYPE | 0.006 |  | 0.004 |  | 1.537 |  | 0.124 |  | -0.0020 | |  | 0.0140 |  |
| Note. INRU= intrusive PTSS, AVOID= avoidance PTSS, COMO= negative changes in cognition and mood PTSS, HYPE = hyperarousal PTSS. Bias-corrected percentile bootstrap confidence intervals. Estimator= Maximum likelihood, Optimization method=NLMINB | | | | | | | | | | | | | | | | | | | |

| Table S4. Total effects of all 4 mediation models. | | | | | | | | |
| --- | --- | --- | --- | --- | --- | --- | --- | --- |
|  | | | | | | | **95% Confidence Interval** | |
|  |  |  | **Estimate** | **Std. Error** | **z-value** | **p** | **Lower** | **Upper** |
| Abuse | → | AVOID | 0.032 | 0.007 | 4.255 | < .001 | 0.017 | 0.046 |
| Abuse | → | INTRU | 0.037 | 0.008 | 4.425 | < .001 | 0.021 | 0.053 |
| Abuse | → | COMO | 0.041 | 0.008 | 5.388 | < .001 | 0.026 | 0.056 |
| Abuse | → | HYPE | 0.040 | 0.008 | 5.134 | < .001 | 0.025 | 0.055 |
| Note. INRU= intrusive PTSS, AVOID= avoidance PTSS, COMO= negative changes in cognition and mood PTSS, HYPE = hyperarousal PTSS. Bias-corrected percentile bootstrap confidence intervals. Estimator= Maximum likelihood, Optimization method=NLMINB | | | | | | | | |

**References**

Derogatis, L. R. (1975). Brief symptom inventory. European Journal of Psychological Assessment.

First, M. B., Williams, J. B. W., Karg, R. S., & Spitzer, R. L. (2016). Structured Clinical Interview for DSM-5® Disorders—Clinician Version (SCID-5-CV).

Pollok, T. M., Kaiser, A., Kraaijenvanger, E. J., Monninger, M., Brandeis, D., Banaschewski, T., … Holz, N. E. (2022). Neurostructural traces of early life adversities: A meta-analysis exploring age- and adversity-specific effects. Neuroscience and Biobehavioral Reviews, 135(February), 104589. https://doi.org/10.1016/j.neubiorev.2022.104589

Spitzer, C., Freyberger, H. J., Stieglitz, R.-D., Carlson, E. B., Kuhn, G., Magdeburg, N., & Kessler, C. (1998). Adaptation and psychometric properties of the German version of the dissociative experience scale. Journal of Traumatic Stress, 11(4), 799–809. https://doi.org/10.1023/A:1024457819547

Thombs, B. D., Lewis, C., Bernstein, D. P., Medrano, M. A., & Hatch, J. P. (2007). An evaluation of the measurement equivalence of the Childhood Trauma Questionnaire—Short Form across gender and race in a sample of drug-abusing adults. Journal of Psychosomatic Research, 63(4), 391–398. https://doi.org/10.1016/J.JPSYCHORES.2007.04.010
